# Effects of Juçara (*Euterpe edulis* Martius) on Health: An Overview of Clinical and Experimental Studies and Call for Action

**DOI:** 10.3390/nu15081809

**Published:** 2023-04-07

**Authors:** Ana P. S. Siqueira, Jéssika M. Siqueira, Mirella P. Lopes, Gustavo D. Pimentel

**Affiliations:** Faculty of Nutrition, Federal University of Goias, Goiânia 74605-080, GO, Brazil; ana.siqueira@ifgoiano.edu.br (A.P.S.S.); jessikanutriufg@gmail.com (J.M.S.); mirelladepaivalopes@gmail.com (M.P.L.)

**Keywords:** functional food, bioactive compounds, supplementation, juçara pulp

## Abstract

Background and aims: Juçara is a fruit of ecological and nutritional importance. Its fruits represent an option for the sustainable use of the plant due to its vulnerability to extinction. Thus, the aim of this review was to analyze clinical and experimental studies and highlight the literature gaps regarding the effects of supplementation with Juçara on health. Methods: For this scoping review, we consulted the Medline (PubMed), Science Direct, and Scopus databases in March, April, and May 2022. Experimental studies and clinical trials published in the last ten years (2012–2022) were analyzed. Data were synthesized and reported. Results: A total of 27 studies were included, 18 of which were experimental studies. Of these, 33% evaluated inflammatory markers associated with fat accumulation. Most of these studies (83%) used pulp in lyophilized form, and the others (17%) involved juçara extract mixed in water. In addition, 78% of the studies showed positive results with respect to the lipid profile, reduction of oncological lesions, inflammation, microbiota modulation, and improvement in obesity and glycemia-related metabolic complications. Nine clinical trials with results similar to those of experimental trials were found. The majority (56%) were chronic (four to six weeks into the intervention), and 44% were acute. Three offered juçara supplementation in the form of juice, four used freeze-dried pulp, two used fresh pulp, and one used a 9% dilution. The dose was fixed at 5 g, but the dilution ranged from 200 to 450 mL. These trials assessed mainly healthy, physically active, and obese individual adults (19–56 years old), and cardioprotective and anti-inflammatory effects, as well as improvement in the lipid profile and prebiotic potential, were observed. Conclusion: Juçara supplementation showed promising results with respect to its effect on health. However, further studies are needed to clarify these possible effects on health and their mechanisms of action.

## 1. Introduction

Juçara (*Euterpe edulis* Mart.) is a palm tree native to the Atlantic Forest that is vulnerable to extinction because of deforestation and intense illegal extraction [1,2,3]. The exploitation chain of this palm has recently been focused on the commercial use of its fruits as a sustainable option [2]. This fruit is physically similar to açaí (*Euterpe oleracea* Mart.) in terms of the nutritional composition and content of bioactive compounds and is sometimes marketed as a single product [2,4,5].

Juçara fruits can be consumed as pulp or as an ingredient in food preparations such as juices, smoothies, and ice creams [4]. In review studies [2,4,5] that aimed to characterize nutritionally juçara fruit, lipids were reported as a predominant nutrient of the fruit, providing high-energy content to the pulp. Concerning the lipid profile, oleic acid is the main fatty acid (>50%); this pulp contains relevant amounts of polyunsaturated fatty acids. The protein content of juçara can be highlighted in comparison with other common fruits. The fiber content of this fruit is around 20–30%, which is relevant. With respect to the micronutrient content, vitamins C and E and minerals such as iron, zinc, calcium, and potassium stand out.

The juçara fruit contains more phenolic compounds than açai fruits [2,6,7]. Of these, anthocyanins are major a major component, with cyanidin as the predominant type [6]. Due to the effects of bioactive compounds on health, several tests were conducted to evaluate the biological activity of the fruit, especially inflammatory activity and antioxidant activity [4,6,7,8]. Thus, the aim of this scoping review was to analyze clinical and experimental studies and highlight the gaps in the literature regarding the effects of supplementation with Juçara on health.

## 2. Methods

### 2.1. Study Design

This scoping review was carried out according to the five-stage structure described by Arksey and O’Malley: step 1: the identification of the research question (How has supplementation with juçara fruit been explored as a functional food in science?); step 2: identification of relevant studies (described in the Search Strategy and Eligibility Criteria); step 3: selection of studies (described in the Processing and Synthesis section); step 4: data mapping (scanning of data about dose, intervention time, sample condition, bioactive compounds content, and main outcomes); step 5: group, summarize, and report the results (Results section). The review structure was verified against the PRISMA Extension for Scoping Reviews checklist.

### 2.2. Search Strategy and Eligibility Criteria

The Medline (PubMed), Science Direct, and Scopus databases were consulted in March, April, and May 2022 to identify relevant literature on the topic (state of the art). The keywords used were “juçara”, “jussara”, or “*Euterpe edulis*”. The inclusion criteria involved publication date restriction, including only manuscripts published in the last ten years (2012–2022) written in any language. Clinical trials (population nonspecific) and experimental studies (population nonspecific) were included. Review studies and those exclusively involving in vitro assays were excluded. Other studies involving supplementation with other fruits of the Euterpe family were excluded. Articles that did not address at least 4 of the variables cited in step 4 were excluded.

Therefore, the focus of this review was supplementation with juçara pulp and the effects of its consumption on health, alternative treatment of diseases, or preventive effects in conditions that lead to diseases. All studies that demonstrated the effects of juçara supplementation in these conditions were eligible, focusing on approaches to anti-inflammatory and antioxidant activity (concept) and its outcomes. Studies with different animal models, participant profiles, and geographic locations (context) were considered.

### 2.3. Processing and Synthesis

All records were analyzed to exclude duplicates. An evaluation of eligible titles and abstracts was performed. Studies that only reported information on the biochemical, chemical, and physical characterization of the fruit were excluded. The authors retrieved and independently evaluated the full texts of eligible articles. Studies were included if they reported at least one outcome on intervention or biological activity of juçara under disease or preventive conditions.

## 3. Results

Data were synthesized and reported in two main sections: clinical trial results and experimental trial results. Of the 27 selected studies, 9 involved clinical trials, and 18 were experimental trials.

### 3.1. Experimental Studies

Eighteen experimental trials are reported in this review (Table 1). Juçara pulp supplementation was used in search of effects on preneoplastic lesions; antioxidant, neuropotential, toxicological, prophylactic, and therapeutic effects on peptic ulcers; dyslipidemia, hepatic steatosis, glucose tolerance, genomic instability, and progression of the cell cycle; antiatherogenic and anti-inflammatory effects (liver, gastrointestinal tract, and colon); and antiobesogenic action.

Of the eighteen studies twelve (67%) centrally addressed the effects of juçara supplementation on inflammatory markers associated with fat accumulation in organs, high-fat diets, and adipose tissue [10,12,14,15,18,19,20,22,23,24,26,27].

Juçara pulp was used in most studies (83%) in lyophilized form mixed in standardized rations [10,11,12,13,14,15,18,19,20,22,23,24,25,26,27]. The other studies (17%) used an aqueous extract obtained by diluting the pulp in water in proportions of 1:2 or 1:3, with one part pulp for two or three parts water administered via gavage [17,21]. The concentrations ranged from 0.25% to 10% and 22.5 mg/kg to 5 g/kg. Intervention times ranged from three days to sixteen weeks (112 days).

Concerning the number of compounds, total phenolics, and anthocyanins, 33% of the studies only one or none of these results [9,11,13,18,21,22]. Only 5% of the studies qualified anthocyanins and showed that cyanidin 3-glycoside and cyanidin 3-rutinoside are predominant in juçara pulp [22].

The other studies (67%) presented the contents of total phenolics and anthocyanins and, in general, related the positive results of the interventions to these compounds and the lipid profile of the pulp. The variation in the content of phenolic compounds was expressed in terms of catechol and gallic acid (data in 100 g of pulp or the proportion of the intervention) and the determined amount of these compounds, ranging from 4.10 to 3976 ± 197.34 (mg GAE/100 g). There was only an expression of phenolic compounds in catechol instead of gallic acid [9]. The others were expressed in gallic acid equivalents. The anthocyanin contents, expressed in similar units, i.e., cyanidin-3-O-glycoside (C3C) and cyanidin-3-rutinoside (C3R), ranged from 47.9 to 301.4 mg C3G/100 g.

The models and sexes of the animals in the different studies also represented a source of variation, with Wistar rats (67% of the studies) C57BL/6 mice (11% of the studies) C57B1/6 mice (5%) and Swiss mice (17% of the studies). Most animals were males (44%) and females represented about 22%, but some studies did not describe the sex of the animals under investigation (34%). 

Based on the reviewed results, it can be noted that with the different tested doses of juçara showed potential in preventing the proinflammatory status and/or modulating inflammation and/or attenuating the effects of inflammation [10,12,15,19,20,22,23,24,27] and in reducing the severity of hepatic steatosis [11,14]. Increased expression of antioxidant enzymes or modulation of the intestinal were also found to be promoted [12,13,18,24].

Supplementation with juçara was also able to reduce preneoplastic colorectal lesions [17], offer protection in the case of induced peptic ulcers [21], provide renal protection [13,18,25], and prevent degeneration and mutagenicity of liver tissue and cells [9,24].

Juçara supplementation also attenuated adverse perinatal effects associated with hypercaloric/hyperlipidic diets [10,12,20,24], improved the glycemic response, and reduced metabolic complications of obesity [15,26]. These studies also reported that supplementation did not generate cytoprotection or antigenotoxicity [9].

### 3.2. Clinical Studies

In our literature search, nine clinical trials were found in which the effect of juçara consumption on the modulation of the inflammatory response, antioxidant defense, prebiotic potential, genotoxicity, and epigenetic regulation, as well as post-consumption metabolic effects of juçara, were reported (Table 2). Four of these trials were acute [9,28,29,30]; in three, the participants ingested a single dose of juçara [28,29,30]; in the other, the intervention lasted for three consecutive days [9].

Another five studies reported on the chronic consumption and epigenetic regulation of juçara—one with a duration of four weeks [33] and the other four with a duration of six weeks [31,32,34,35]. Four of the trials had a crossover design with washout ranging from seven days [28,29,30] to four weeks [33], and another four studies comprised randomized controlled, single or double-blind interventions [31,32,34,35]. There is one study have no described the design of the study [9].

Juçara pulp was the raw material used in all tests, but there was variation in the level of processing and the dose used. Among the total of tests, juçara juice (commercial product) was used in the intervention in five [9,28,29,30,33], four studies used freeze-dried pulp [31,32,34,35].

The doses also varied between the tests; when the pulp was lyophilized, the dosage was 5 g in all studies [31,32,34,35], and when fresh pulp, diluted pulp, or juice was used, the dose ranged from 200 to 450 mL, with higher doses of 250 mL, 300 mL (9%), and 450 mL corresponding to acute studies [9,28,29,30,33].

As the doses and level of processing varied, as well as the origin of the pulp, the contents of total phenolic compounds and anthocyanins of the sample also varied. In acute trials [27,28,29,30], the levels of total phenolic compounds provided by the supplementation ranged from ~350 mg to 1992 mg GAE, and those of anthocyanins range from ~185 to 2033 mg (total monomeric anthocyanins). In chronic consumption assays [31,32,33,34,35], levels of phenolic compounds varied from ~207 to 1300 mg GAE, and those of anthocyanins ranged from ~131 to 626 (total monomeric anthocyanins).

The sample sizes ranged from 6 to 35 participants; only two studies included only men [29,30], and the others included both sexes. Among the total number of studies, three included healthy individuals [27,28,33], two studies included healthy and physically active individuals [29,30], and four included individuals in grades I and II obesity [31,32,34,35].

All studies included adults ranging in age from 19 to 56 years. Study outcomes show that acute interventions of juçara supplementation before high-intensity intermittent physical exercise or high-intensity interval training (HIIE or HIIT) attenuated the inflammatory response after the session and reduced fatigue, as evaluated by inflammatory and oxidative stress biomarkers [29,30].

Other studies with healthy individuals [28,33] evaluated antioxidant defense, lipid peroxidation, and effects on glucose and reported a positive effect of juçara consumption on antioxidant status and oxidative damage at the cellular level of these individuals due to increases in FRAP (ferric-reducing antioxidant) and GPx (Glutathione peroxidase), the latter exhibiting maximum activity two hours after the intervention.

Juçara consumption seems useful for antioxidant protection and modulation strategies due to increased CAT (catalase) and HDL-c (high-density lipoprotein cholesterol) levels [33]. Juçara pulp does not offer antigenotoxic effects under the conditions of the analyzed study [9].

Studies with interventions in obese patients [31,32,34,35] describe the potential of juçara consumption for inflammatory modulation, prebiotic and metabolic effects, and epigenetic regulation. Still, juçara has the potential to be used as a tool against the proinflammatory state in obesity [35].

Studies suggest a cardioprotective and anti-inflammatory effect of juçara supplementation due to positive the reduction of body fat, an increase in HDL-c, and a twofold increase in the level of adiponectin [34]. Another result was that juçara supplementation improved the serum fatty acid profile, modulating epigenetic markers [31] in monocytes of obese individuals. Furthermore, researchers reported the prebiotic potential of juçara pulp, highlighting its bifidogenic effect and the induction of increased acetate excretion [32].

## 4. Discussion

The aim of this scoping review was to analyze clinical and experimental studies and highlight the gaps in the literature regarding the effects of supplementation with juçara on health. Following the PRISMA guidelines, we identified 27 articles that met the inclusion criteria using juçara in a prophylactic or therapeutic way in diverse health conditions.

### 4.1. What Is Attributed to the Beneficial Effects of Juçara?

In general, the studies cited in this review justify the use of juçara pulp due to its lipid profile, its richness in unsaturated fats, high levels of dietary fiber, and the presence anthocyanins in the pulp. It is possible to notice that, in general, these studies attribute benefits to mechanisms related to reduced inflammation, oxidative stress, and lipid flow in different contexts.

#### 4.1.1. Anti-Inflammatory Effect

The inflammation-reducing effect reported in this study is attributed to flavonoids and their action in the lipoxygenase and cyclooxygenase pathways, in addition the reduction in inflammatory cytokine expression and TLR4 signaling. In this context, juçara seems to reduce TNF-α and COX2 expression [24], controlling cell migration and the mobility of inflammatory mediators [10].

The polyunsaturated fatty acids present in the pulp were also considered to have a potential impact on the inflammatory response of NF-κB mediated by TLR4 [12,31]. Data [12,19,31,34,35] show that using juçara can reduce phosphorylation of the NF-κBp50 subunit mediated by TLR4 activity through the MYD88-dependent pathway.

#### 4.1.2. Elimination of Free Radicals

The antioxidant activity of juçara pulp is attributed to phenolic compounds, mainly anthocyanins. The studies analyzed in this review evaluated the redox status, especially for MDA, CAT, SOD, GST, GPx, and GSH, enzymes involved in oxidative mechanisms. 

The effect of juçara consumption on oxidative stress seems to be due to the indirect action of anthocyanins, increasing antioxidant defenses through different mechanisms: increased activity of enzymes such as SOD, GPx, and GSH; activating genes encoding these enzymes; or reducing ROS formation [11,14,17,18,22,33]. In addition, anthocyanins from juçara may induce the endogenous antioxidant enzymatic system, neutralizing reactive metabolites and modulating oxidative stress [14].

### 4.2. What Are the Gaps Regarding the Effects of Supplementation with Juçara on Health?

#### 4.2.1. Experimental Design

In short, the available studies are predominantly experimental tests using rats or mice performed when the investigation of human beings is not feasible. Results from these studies are incipient, although several health conditions have been evaluated because experimental studies are limited regarding the extrapolation of results to human beings due to differences between species, metabolic mechanisms, and dosages that may invalidate generalizations [36,37]. Most clinical studies analyzed herein have robust designs, such as crossover or placebo-controlled single or double-blind designs. Study design is important to obtain consistent results to be used in clinical practice as both therapeutic and preventive measures.

Two studies with different levels of evidence (one clinical and another experimental) evaluated the supplementation of juçara in obesity. However, the experimental study evaluated the effects of juçara supplementation on energy homeostasis and metabolic complications in obesity [26], whereas the clinical study investigated changes in the serum fatty acid profile and the ability of juçara pulp to modulate epigenetic markers in the monocytes of obese adult humans [31]. Thus, it is notable that there was no confirmation of the results of these studies.

In our view, the experimental studies in which juçara supplementation was investigated and the clinical trials studied in this review do not have a clear continuity relationship, that is, these studies do not complement each other on the same themes. Therefore, it is not possible to verify the construction of scientific knowledge about the possible benefits of juçara supplementation, which creates a gap in the literature.

The relationship between experimental studies and clinical trials is important because they function as steps in the construction of scientific knowledge with the objective of practical application.

#### 4.2.2. Juçara Pulp and Supplementation Type

The pulp composition of bioactive compounds (total phenolic compounds and anthocyanins) is highly variable due to agronomic and environmental issues. Methodologies for the determination and unit expression of these compounds must also be considered. This variation of compounds makes it difficult to standardize the use of pulp for the future interventions. However, it is noteworthy that the doses are physiological, especially in clinical trials, which is characteristic of interventions that can be performed at the diet level and not necessarily at the pharmacological level. 

Another point to be addressed with respect to the difference between experimental and clinical studies is that in the experimental studies, there is a diversity in the supplementation of juçara (different pulp concentrations, induced high-fat diets, and changes in pulp lipid composition). However, in clinical studies, there was no change in the pulp composition and dietary intervention.

The dose and level of supplementation varied in both experimental and clinical studies. The lack of standardization in supplementation is harmful because it reduces the reproducibility of studies and inferences that support new studies with other outcomes. We emphasize that part of the dose variations between studies is due to metabolic differences and varying proportions of the body surface of humans and animals. Even so, it seems necessary to point out that some studies converge on doses between 50 and 100 g of fresh pulp/day, which is equivalent to between 5 and 10 g of dehydrated pulp.

With respect to the processing level, the use of lyophilized (or dehydrated) products seems to predominate, which is possibly due to the practicality of handling and storing the material. Furthermore, there is a lack of understanding of the indication of an intervention, whether acute or chronic, as well as the intervention time.

#### 4.2.3. Bioavailability and Human Participants 

The reviewed studies did not assess the bioaccessibility and/or bioavailability of the compound with which the events are associated.

Another point to be discussed is the age group of the participants in the clinical trials, which seems to be homogeneous, as the evaluated individuals were, in all cases, adults. On the other hand, the difference between the use of juçara in female and male metabolism has not been explored.

## 5. Conclusions

The aim of this scoping review was to analyze clinical and experimental studies and highlight the gaps in the literature regarding the effects of supplementation with juçara on health. Overall, the results from the reviewed studies support two conclusions. First, juçara supplementation showed promising results with respect to its effect on health in different conditions associated with reduced inflammation effect and oxidative stress due to bioactive compounds, especially anthocyanins. Second, studies did not elucidate the mechanisms of action and bioavailability of anthocyanins, and no specific dose has been established for supplementation.

Therefore, more studies are needed, especially clinical trials, to clarify the real effects of juçara supplementation and address the mechanisms of action, given the reported effects, in order to allow for standardization of the supplementation dose to be used in different health contexts and diseases in humans.

## Figures and Tables

**Table 1:** Juçara pulp supplementation results in search of effects on preneoplastic lesions; antioxidant, nephroprotective, toxicological, prophylactic, and therapeutic effects on peptic ulcers; dyslipidemia, hepatic steatosis, glucose tolerance, genomic instability, and cell cycle progression cellular; as well as antiatherogenic and especially anti-inflammatory effects, in studies with rats, and mice.

Authors /Year	Pulp Processing/Dose	Intervention Time	Sample/Condition	Phenolics (FT) and Anthocyanins (ANT) of the Portion or in 100 g	Outcome/Results
Felzenszwalb et al., (2013) [9]	Aqueous extract. Doses (mg/kg): 22.5, 45, 90, and 180	3 consecutive days	25 male Wistar rats	FT: 15 mg/mL (catechol equivalent); ANT: not reported	Toxicological and pharmacological evaluation ↑ Levels of ALT and glucose in animals supplemented with a dose of 180 mg/kg of juçara extract and only glucose in animals supplemented with 90 mg/kg. ↑ Micronucleated polychromatic erythrocytes in animals supplemented with 45–180 mg/kg doses; ↑ mitotic index was detected in animals supplemented with 90 and 180 mg/kg doses. There was NO positive genotoxic response in the groups supplemented with juçara. NO DNA lesions were observed at the concentrations used in the study.
Morais et al., (2014) [10]	Freeze-dried juçara pulp. Dose: 5 g/kg in the control or diet with hydrogenated vegetable fat	Supplementation occurred from the first day of pregnancy and throughout the gestation period. The offspring were evaluated for 21 days after birth.	Pregnant Wistar rats were exposed to trans fatty acids and offspring at 21 days of age.	FT: 415 ± 22.3 mg GAE/100 g; ANT: 239.16 ± 7.6 mg C3R/100 g	Effect on the proinflammatory state in the intestinal tract of 21-day-old offspring. ↓ Levels of total cholesterol; glucose; and the expression of TNF-αR, TLR4, IL6-R, IL-6, TNF-α in animals fed a high-fat diet. ↓ Triacylglycerol levels compared to the control group and the group fed a high-fat diet. ↑ *Lactobacillus* spp. genomic DNA levels in the colon of offspring supplemented with juçara compared to the high-fat diet group. ↑ IL-10 in the control diet + juçara group compared to the control group and the group fed a high-fat diet supplemented with juçara.
Cardoso et al., (2015) [11]	Lyophilized pulp. Doses: 2%, 6%, and 10%	75 days of evaluation	60 mice (10-C57BL/6 and 50-ApoE mice (–/–))	FT: none; ANT: 301.4 mg C3G/100 g; average daily consumption of anthocyanins reported per animal: 0.45 mg (2%), 1.40 mg (6%), and 2.29 mg (10%)	Effects of pulp consumption on lipid metabolism and steatosis in knockout apoE rats (–/–) ↓ HDL cholesterol fraction in the group supplemented with 10% of juçara compared to other doses and controls. ↓ Non-HDL fraction in the groups supplemented groups with 10% the group supplemented with a 2% dose and controls. ↓ Triglyceride levels with 6 and 10% compared to controls. ↓ SOD levels in the group supplemented with 6% juçara pulp. ↓ Accumulated lipid droplets in liver tissue in animals supplemented with 6 and 10% juçara pulp.
Morais et al., (2015) [12]	Freeze-dried juçara pulp. Dose: 0.5%	Supplementation during pregnancy and lactation	Pregnant/lactating Wistar rats and 21-day-old offspring	FT: 415.1 ± 22.3 mg GAE/100 g of pulp; ANT: 262.4 ± 8.6 mg C3G/100 g of pulp	Prevention of adverse effects induced by trans fatty acids and effects in a proinflammatory state. ↓ IL-6 levels in retroperitoneal white adipose tissue of pulps fed a high-fat maternal diet. ↓ IL-6 and TNF-α in the liver of pups fed a maternal diet supplemented with juçara compared to pups from high-fat and control dams. ↑ IL-10 in retroperitoneal white adipose tissue of pulps fed a high-fat maternal diet relative to the controls. ↑ IL10/TNF-α ratio in retroperitoneal white adipose tissue of puppies compared to the control group. ↑ IL10/TNF-α ratio in the liver of pulps from mothers fed a high-fat diet. ↓ NFkB activation, MyD88 and p-NFkB p65 expression, and TNFR1 expression in the liver of puppies in the high-fat diet group. ↑ Bifidobacterium spp. level and ZO-1 expression levels in the colon of pulps with supplemented high-fat diet mothers related to the high-fat diet controls.
Novello et al., (2015) [13]	Lyophilized pulp. Doses: 2% and 6%	75 days of supplementation	40 Mice (C57BL/6) with apolipoprotein E deficiency (ApoE–/–) and 10 knockout E C57BL/6 mice	FT: not reported; ANT: 24,714 g.kg^−1^	Antiatherogenic and antioxidant activity ↓ LDL content, cholesterol/HDL, and LDL/HDL ratios in the supplemented groups compared to the control. ↓ Glucose content in the supplemented groups relative to the control. No hepatotoxic or nephrotoxic effects were evidenced in association with juçara supplementation. The use of juçara did not affect the size of atherosclerotic plaques. ↓ CAT and SOD levels in the supplemented groups compared to the positive control.
Freitas et al., (2016) [14]	Lyophilized pulp. Dose: integral, 5 and 10%; degreased, 5 and 10%	4 weeks of supplementation	42 Male Wistar rats with high-fat-diet-induced non-alcoholic fatty liver disease	FT: 4.10 ± 0.13; 4.95 ± 0.07 (mg GAE/g); ANT: 2130 ± 114; 3121 ± 139 (mg GAE/g)	Effect on morphofunctional parameters in the injured liver. ↓ Pathognomonic characteristics of steatosis in animals supplemented with 10% defatted pulp compared to the high-fat diet group. ↓ Inflammatory infiltrates in liver tissue in animals treated with 10% wholegrain pulp compared to the high-fat diet group. ↓ Malonaldehyde (MDA) levels and SOD content in animals supplemented with whole/defatted juçara extracts at all doses. ↓ CAT and GST in animals treated with 10% whole or defatted juçara pulp. There was no difference between groups in C-reactive protein, AST, ALT, triglycerides, or HDL levels.
Oyama et al., (2016) [15]	Lyophilized juçara pulp. Doses: 0.5% and 2%	2 weeks of supplementation	66 Swiss mice	FT: 415.1 ± 22.3 mg GAE/100 g; ANT: 239.16 ± 7.6 mg C3R/100 g; data from [16]	Effect of juçara supplementation on glucose tolerance and adipose tissue inflammation ↑ Blood glucose and area under the curve in animals supplemented with 2% juçara fed a commercial diet compared to the control and the 0.5% group. ↓ Blood glucose (up to 60 min) in the area under the curve in animals fed a high-fat, high-calorie diet supplemented with 0.5% compared to the control and the 2% supplement group. ↑ Relative body mass and mesenteric adipose tissue in animals fed a commercial diet with 2% juçara. The addition of juçara in the group fed a high-fat, high-calorie diet did not affect the relative mass or adiposity of the animals. ↑ Total cholesterol in animals treated with 2% juçara and fed a commercial diet compared to the control. ↓ Adiponectin levels in animals fed a commercial diet supplemented with 0.5% compared to the control. ↑ Insulin and adiponectin levels in the animals fed a high-fat, high-calorie diet supplemented with 2% juçara compared to the control. ↑ TNF-α IL-6 and IL-10 concentrations in epididymal tissue of animals fed a supplemented with 0.5% juçara pulp; and increase of IL-6 only in animals supplemented with 2% juçara.
Dos Reis et al., (2019) [17]	Aqueous extract in a 1:2 pulp/water ratio. Dose: 25–45 mL/12 h (0.3%)	14 days of supplementation	21 female rats (Rattus norvegicus) with colorectal carcinogenesis induced by 1,2-dimethylhydrazine (DMH)	FT: 1226.39 ± 21.08 (mg GAE/100 g); ANT: 298.86 ± 27.68 (mg C3G/100 g). Note: It is estimated that in 12 h, 1.23 mg of total phenolic compounds was consumed.	Effect of juçara pulp supplementation on the development of preneoplastic lesions. ↓ Total number of aberrant crypt foci relative to the DMH+/juçara− group. ↑ Expression and content of SOD1 in the colorectal mucosa relative to the juçara supplementation.
Freitas et al., (2017) [18]	Freeze-dried whole pulp: 5%; Freeze-dried whole pulp: 10%; Pulp. Degreased: 5% Pulp. Degreased: 10%.	20 days of supplementation	60 Wistar rats (Rattus norvergicus albinus) fed a cafeteria diet or commercial diet	No description or reference	The antioxidant and toxic to kidney and heart tissue. NO effects on kidney tissue were found. ↑ CAT expression in animals supplemented with 5 and 10% whole meal pulp and 10% defatted cafeteria diet compared to the control group. ↑ Glutathione S-transferase in animals supplemented with 10% defatted lyophilized pulp or not in a commercial diet.
Santamarina et al., (2019) [19]	Lyophilized pulp. Doses: 0.25% and 0.5%	7 days of supplementation	27 male Wistar rats	FT: 3976.1 ± 197.34 mg/100 g; ANT: 2663.7 ± 76.2 mg/100 g	Prevention of deleterious effects of inflammation and fat accumulation in the liver induced by a high-fat diet. ↑ Triacylglycerol and AST levels in animals supplemented with 0.5% juçara pulp with a high-fat diet compared to the control. AST levels in animals supplemented with 0.25% juçara in a high-fat diet were similar to the control. ↑ IL-6 in retroperitoneal tissue in the groups supplemented with 0.25 and 0.5% juçara with a high-fat diet and TNF-α in the group supplemented with 0.5% in a high-fat diet compared to the control. ↓ IL-10 in retroperitoneal tissue and liver of animals supplemented with 0.5% juçara in a high-fat diet compared to the control. The Expression of TRAF-6 in retroperitoneal tissue in animals supplemented with 0.25% juçara was similar to the control. ↓ Positive and strong correlation between the high-fat control and high-fat diet groups with 0.5% juçara supplementation in terms of visceral adiposity.
Argentato et al., (2017) [20]	Freeze-dried juçara pulp. Dose: 0.5%	Supplementation during pregnancy and lactation up to 21 days after birth of offspring	Pregnant/lactating Wistar rats and 21-day-old offspring and male pups fed a diet high in hydrogenated fat	FT: 415.1 ± 22.3 mg/100 g; ANT: 262.4 ± 8.6 mg/100 g	UCP-1 expression and proinflammatory state. ↓ Weight gain in animals fed a commercial diet supplemented with juçara relative to the control group. ↓ Glucose and triglyceride content in the high-fat diet group compared to the high-fat diet + juçara group. ↑ TNF-α levels in the high-fat diet + juçara group compared to the hyperlipidic control and the commercial diet + juçara group. ↑ IL-10 levels in the commercial diet + juçara group relative to the control and the group fed a high-fat diet + juçara. ↑ IL10/TNF-α ratio in the commercial diet + juçara group relative to the group fed a high-fat diet + juçara. ↑ UCP1 protein levels in the high-fat diet + juçara group relative to the high-fat diet control and the group fed a commercial diet + juçara.
Torres et al., (2018) [21]	Aqueous extract (1:3 g pulp/mL of water). Dose: 2 g/kg	3 days of supplementation (before prophylactic or after ulcer induction therapy)	16 male Wistar rats with induced peptic ulcers	No information provided	Prophylactic activity and antiulcerogenic therapy. ↓ In cases of ulcers compared to the control group (with omeprazole). ↓ Microscopic characteristics of necrosis and fibrosis in the juçara group, similar to the omeprazole group. Protection against gastric ulcer formation compared to the negative control and neocapillary group.
Freitas et al., (2018) [22]	Whole freeze-dried pulp (10%). Defatted freeze-dried pulp (10%)	50 days	32 Wistar rats (Rattus norgicus)	Only anthocyanins were identified, with the most expressive peak corresponding to cyanidin-3-glucoside and cyanidin-3-rutinoside.	Redox status and expression of inflammatory mediators. ↓ Total cholesterol levels in the group that received defatted pulp relative to the control and the group supplemented with whole pulp. ↓ Levels of malonaldehyde in the groups supplemented with juçara. ↓Levels of CAT, GST, and SOD in the group supplemented with defatted pulp relative to the control and the group supplemented with whole pulp. ↓ Levels of proinflammatory mediators IL-17, IFN, and TNF-α in the group supplemented with defatted pulp and reduced anti-inflammatory markers IL-4 and IL-10.
Santamarina et al., (2018) [23]	Lyophilized juçara pulp. Doses: 0.25% and 0.5%	7 days of supplementation	27 male Wistar rats	FT: 415.1 ± 22.3 mg/100 g; ANT: 262.4 ± 8.6 mg/100 g. Note: 0.5% represents the average of 6 mg of anthocynins/mice/day.	Prevention of deleterious effects induced by a high-fat diet. ↑ Weight gain in the group supplemented with 0.5% relative to the control. ↑ Triacylglycerol content in the group supplemented with 0.5% compared to the 0.25% group. ↓ TNF -α in the groups supplemented with juçara relative to the control. ↓ IL-6 in the group supplemented with 0.25% compared to the control. ↑ NF-kBp50 in the group supplemented with 0.5% of juçara relative to the control.
Argentato et al., (2019) [24]	Lyophilized juçara pulp. Dose: 0.5%	From the first day of pregnancy and during lactation	Wistar rat puppies fed a diet containing trans fatty acids	FT: 415.1 ± 22.3 mg GAE/100 g; ANT: 239.16 ± 7.6 mg C3R/100 g of pulp (data from [16])	Effect of juçara supplementation on maternal trans fatty acids in the liver of 21-day-old pups. In animals treated only with juçara, no histopathological differences were found relative to the negative control. In the group exposed to trans and juçara fatty acids, mild changes in liver tissue were observed. ↓ Number of micronucleated hepatocytes in the group exposed to trans fatty acids + juçara relative to the group exposed only to trans fatty acids. ↓ TNF-α, COX-2, and Ki-67 levels in the supplemented group compared to that exposed to trans fatty acids.
Cardoso et al., (2020) [25]	Lyophilized juçara pulp. Doses: 100, 200, and 400 mg/kg	5 days before induction of nephropathy	70 Swiss male mice with induced nephropathy	FT: 811 ± 16.7 mg GAE/g; ANC: 181.25 ± 5.36 mg C3G/100 g	Effect on prevention of acute kidney injury. Animals supplemented with juçara had creatinine levels Equal to those of the negative control group. ↑ Renal protection in the group treated with 100 mg/kg of juçara, as indicated by the severe urea levels better than the group treated with n-acetylcysteine. ↑ Protein oxidation in the group supplemented with 400 mg/kg of juçara compared to the control. ↑ Preservation of renal cell structure in the group supplemented with 100 mg/kg, similar to the group treated with n-acetylcysteine. No toxic effects were found at the doses of juçara used in this study.
Barthichoto et al., 2021) [26]	Lyophilized juçara pulp. DoseL: 0.5% (5 g of pulp in 1 kg of standardized diet)	16 weeks	n = 8–10 12-week-old male C57B1/6 mice	FT: 415.1 ± 22.3 in (GAE) 100 g; ANT: 47.9 ± 1.5 mg C3G/100 g f.m) and 179.6 ± 5.7 mg C3G/100 g f.m. Note: 0.5% of juçara in the diet is equivalent to 3.3 mg of anthocyanins/kg/day.	Effect on complications associated with obesity. Animals supplemented with juçara on a control diet gained weight; animals supplemented juçara on a high-fat diet lost weight despite similar energy intake and expenditure between the high-fat diet groups. Similar UCP expression in the group supplemented with a high-fat diet to that the control group on a high-fat diet. Severe insulin resistance was partially recovered with juçara supplementation. Less fat accumulation in hepatocytes in the supplemented group than in the control, both on a high-fat diet.
Silva et al., (2021) [27]	Lyophilized juçara pulp. Doses: 0.50% + high-fat diet and 2% + high-fat diet	16 weeks of supplementation	60 male Swiss mice	FT: 415.1 ± 22.3 mg GAE/100 g; ANT: 239.16 ± 7.6 mg/100 g. Note: characterized in a previous study [16].	Effect on systemic, tissue, and local (colon) inflammation. Similar levels of LPS and TNF-α between the groups supplemented with juçara and those on a hypercaloric diet. ↑ TNF-α in epididymal fatty tissue in the group supplemented with 2% juçara compared to the group supplemented with 0.5% juçara pulp.

FT: total phenolic compounds; ANT: total anthocyanins; GAE: gallic acid equivalent; C3G: cyanidin-3-O-glucoside; C3R: cyanidin-3-O-rutinoside; DMH: 1,2- dimethylhydrazine; SOD: superoxide dismutase; CAT: catalase; AST: aspartate transaminase; IL-6: interleukin-6; TNF-α: tumor necrosis factor α; IL-10: interleukin-10; Traf-6: tumor necrosis factor receptor-associated factor 6; MDA: malondialdehyde; GST: glutathione-s-transferase; ALT: alanine aminotransferase; HDL: high-density lipoprotein; IL6-R: interleukin-6 gene expression; TNF-αR: tumor necrosis factor α gene expression; TLR-4: Toll-like receptor 4; DNA: deoxyribonucleic acid; COX-2: anti-COX2 antibody; Ki-67: anti-Ki-67 monoclonal antibody; UCP: uncoupling protein 1; IFN: gamma interferon; IL-17: interleukin-17; IL- 4: interleukin-4; LPS: lipopolysaccharide; LDL: low-density lipoprotein; NF-kB: nuclear factor kappa-B; MyD88: myeloid differentiation primary response 88; *p*-NFKB p 65: nuclear factor NF-kappa-B p65; TNF-R1: tumor necrosis factor receptor 1; ZO1: zonula occludens-1; NFKB p 50: nuclear factor NF-kappa-B p50.

**Table 2:** The inflammatory modulation, antioxidant defense, prebiotic potential, genotoxicity, epigenetic regulation, and metabolic effects after consumption of juçara in clinical trials according to the scientific literature.

Authors/Year	Clinical Design	Pulp Processing/Dose	Intervention Time	Sample/Condition	Phenolics (FT) and Anthocyanins (ANT) of the Portion or in 100 g	Outcome/Results
Felzenszwalb et al., 2013) [9]	Not described	Juçara juice (9%) commercial product. Dose: 300 mL	3 consecutive days	*n* = 6; sex = both; age = 25–35; years; healthy	FT: 15 mg/mL (catechol equivalent); ANT: not reported	Genotoxicity No positive pre- or post-treatment genotoxicity response was identified.
Cardoso et al., (2015) [28]	Randomized crossover	Juçara juice (commercial product). Dose: 450 mL	One-time (acute study assessments between 0 and 4 h postintervention); washout for 7 days	*n* = 11; sex = both; age = 24–30 years; healthy	FT: 1992.1 ± 59.1 mg in 450 mL; ANT: 2033.7 C3G mg ± 28.1 in 450 mL	Antioxidant activity and lipid peroxidation The peak of antioxidant activity (FRAP) occurred in the first hour after ingestion of juçara juice. A positive correlation was found between antioxidant activity (FRAP) and uric acid content. Maximum GPx activity occurred after two hours of juçara consumption. Supplementation with juçara did not affect GSH levels. Supplementation positively correlated with the time for lipid peroxidation, and the greatest difference between treatments occurred after two hours of ingestion of juçara juice.
Santamarina et al., (2018) [31]	Randomized double-blind controlled placebo	Lyophilized juçara pulp. Dose: 5 g	6 weeks	*n* = 27; sex = both; age = 41–48 years; obese (BMI between 33 and 36 kg/m^2^)	FT: 415.1 ± 22.3 mg in 100 g of fresh matter; ANT: 262.4 ± 8.6 mg in 100 g of fresh matter	Changes in fatty acid profile and epigenetic regulation in obesity ↓ Sum of saturated fatty acids in the supplemented group relative to the control. ↑ Sum of monounsaturated fatty acids in the supplemented group relative to the control. ↑ Omega-3 levels relative to baseline in the supplemented group. ↓ Omega-6/3 ratio in the supplemented group compared to baseline. ↓ DNM1, DNMT3a, DNMT3b, and MeCP2 pre-treatment levels without LPS in the supplemented group versus placebo. ↑ Post-treatment DNM1, DNMT3a, DNMT3b + LPS, and MeCP2 levels in the supplemented group versus the placebo group. ↑ MeCP2 expression with or without LPS in the supplemented group versus the placebo group. Juçara treatment and oleic acid concentration are predictors of MeCP2 mRNA levels.
Copetti et al., (2020) [29]	Single-blind randomized crossover	Juçara juice. Dose: 250 mL	1 h before HIIT exercise; washout for 7 days	*n* = 15; gender = male; age = 23–28 years; healthy and physically active	FT: 350 mg ± 17.50 in 250 mL; ANT: 185 C3G mg ± 7.50 out of 250 mL	Reduction in oxidative stress and fatigue after HIIT exercise. There was NO significant difference between groups and over time for the ratio of GSH/GSSG, PC, GPx, SOD, and CAT. ↓ Level of fatigue of the supplemented group relative to the control. ↑ GSSG in the supplemented group versus control immediately after exercise and GSH one hour after exercise. ↑ Total phenolic compounds and uric acid after a 1 h exercise session in the supplemented group.
Jamar et al., (2020) [32]	Randomized double-blind controlled placebo	Lyophilized juçara pulp. Dose: 5 g	6 weeks	*n* = 35; sex = both; age = 39–56 years; obese (BMI between 30 and 39 kg/m^2^)	FT: 207.55 mg in 5 g of pulp; ANT: 130.7 mg in 5 g of pulp	Modulation of commensal bacteria and SCFA production. ↑ Levels of fecal acetate (SCFA) and abundance of *A. muciniphila*, *Bifidobacterium* spp., and *C. coccoides* in the group supplemented with juçara relative to baseline and the control.
Liz et al., (2020) [33]	Single-blind randomized crossover	Juçara juice (commercial product). Dose: 200 mL/day (100 mL twice a day)	4 weeks; 4-week washout	*n* = 30; sex = both; age = 19–48 years; healthy	FT: 1300.17 ± 91.39 in 200 mL; ANT: 626.57 C3G mg ± 17.97 in 200 mL	Effects on glucose, lipid profile, and oxidative stress ↑ HDL and CAT content after juçara supplementation.
Jamar et al., (2020) [34]	Randomized double-blind controlled placebo	Lyophilized juçara pulp. Dose: 5 g/equivalent to 50 g of fresh pulp	6 weeks	*n* = 35; sex = both; age = 39–56 years; obese (BMI between 30 and 39 kg/m^2^)	FT: 207.55 mg in 5 g of pulp; ANT: 130.7 mg in 5 g of pulp	Antiobesogenic potential with emphasis on metabolic parameters. ↑ Fat-free mass in the group supplemented with juçara relative to baseline. ↓ Body fat, total cholesterol, LDL-c, HDL, triacylglycerol, TGA/HDL ratio, adiponectin, and leptin/adiponectin ratio in the juçara-supplemented group compared to baseline. ↑ Amount of body fat in the placebo group compared to the supplemented group. ↓ HDL, adiponectin, and adiponectin/homa-IR ratio levels in the placebo group compared to the supplemented group.
Santamarina et al., 2020) [35]	Randomized double-blind controlled placebo	Lyophilized juçara pulp. Dose: 5 g	6 weeks	*n* = 27; sex = both; age = 41 to 48 years; obese (BMI between 33 and 36 kg/m^2^)	FT: 415.1 ± 22.3 mg in 100 g of fresh matter; ANT: 262.4 ± 8.6 mg in 100 g of fresh matter	Inflammatory status ↑ TLR4 following pretreatment LPS stimulation in the supplemented versus placebo subjects. ↓ Post-treatment TLR4 with juçara supplementation with or without LPS stimulation. ↓ IL-6 levels in the supplemented group compared to the placebo group. ↑ Post-treatment IL-10 levels in the juçara group versus the placebo group. ↑ Ob-R post treatment in the juçara with LPS group versus the placebo with LPS group. ↓ MYD88, which was shown to be modulated in the juçara supplementation with LPS group compared to the placebo group post treatment. ↓ pIkk α and β post treatment in the juçara group versus the placebo without LPS group post treatment. ↑ MCP-1 levels in the no-LPS group supplemented with juçara pretreatment compared to placebo group.
Mendes et al., (2021) [30]	Randomized crossover single-blind	Juçara juice (commercial product). Dose: 250 mL	1-h before HIIE (acute study); washout for 7 days	*n* = 15; gender = male; age = 23–28 years; healthy and physically active	FT: 350.0 ± 17.5 mg/GAE in 250 mL; ANT:186.0 ± 7.5 mg in 250 mL	Inflammatory adaptive response after HIIE ↑ IL-6 over time in both groups. ↓ TNF-α levels 30 min after exercise in the control group and 60 min after exercise in the supplemented group. ↑ Cortisol levels in the control group after 60 min compared to the supplemented group. There was no difference in r PPO Sprint between groups (maximum performance).

GAE: gallic acid equivalent; HIIE: high-intensity intermittent exercise; IL-6: interleukin-6; TNF-α: tumor necrosis factor; IL-10: interleukin-10; rPPO Sprint: relative difference from peak power output sprint test; HITT: high-intensity interval training; GSH: glutathione; GSSG: oxidized glutathione; PC: protein carbonyl; Gpx: glutathione peroxidase; SOD: superoxide dismutase; HDL: high-density lipoprotein; CAT: catalase; FRAP: ferric-reducing antioxidant; BMI: body mass index; TLR4: Toll-like receptor 4; LPS: lipopolysaccharide; Ob-R: leptin receptor; MYD88: myeloid differentiation primary response 88; PIKK α and β: phospho-IKK alpha/beta; MCP-1: monocyte chemoattractant protein-1; SCFA: short-chain fatty acid; LDL-c: low-density lipoprotein cholesterol; TGA/HDL: ratio triacylglycerol/high-density lipoprotein; Homa-IR: homeostatic model assessment for insulin resistance; DNM1: dynamin-1; DNMT3a: DNA methyltransferase 3 alpha; DNMT3b: DNA methyltransferase 3 beta; MeCP2: methyl CpG fatty acid protein 2; mRNA: messenger RNA.

## Data Availability

The data presented in this study are available on request from the corresponding author.
